# Leptin-dependent co-regulation of bone and energy metabolism

**DOI:** 10.18632/aging.100100

**Published:** 2009-11-05

**Authors:** Vijay K. Yadav, Gerard Karsenty

**Affiliations:** Department of Genetics and Development, Columbia University Medical Centre, New York, NY 10032, USA

**Keywords:** leptin, serotonin, bone, appetite, energy metabolism

## Abstract

The adipocyte-derived hormone leptin inhibits appetite and bone mass accrual. To fulfill these two functions leptin requires the integrity of hypothalamic neurons but not the expression of its receptor, ObRb on these neurons. These results suggested that leptin acts first elsewhere in the brain to mediate these functions. However, this neuroanatomical site of leptin action in the brain remained elusive. Recent mouse genetic, electrophysiological and neuroanatomical studies provide evidence that leptin inhibits appetite and bone mass accrual through a two-step pathway: it decreases synthesis and the release by brainstem neurons of serotonin that in turn targets hypothalamic neurons to regulate appetite and bone mass accrual.

Skeleton in vertebrates serves multiple mechanical, hematopoietic and endocrine functions [1,2]. In order to perform its functions properly, the skeleton continuously renews itself through a homeostatic process known as bone remodeling [1,3]. Bone remodeling occurs constantly and simultaneously in numerous parts of skeleton spread throughout the body and requires considerable inflow and utilization of energy [4]. Any perturbance in energy homeostasis of the body can therefore result in dramatic changes in skeletal metabolism [5,6]. For example, obesity or high body mass index often reduces fracture risk, whereas on the other hand anorexia enhances it [5,6]. These observations provide clinical evidence that bone and energy metabolism are balanced with each other and are likely co-regulated.

Serotonin (5-hydroxytryptamine) is a biogenic amine that functions both as a neurotransmitter in central nervous system and as a hormone in the periphery where most of it (95%) is produced [7,8]. Serotonin is generated through an enzymatic pathway in which L-tryptophan is converted into L-5OH-tryptophan by an enzyme called tryptophan hydroxylase (Tph); this inter mediate product is then converted to serotonin by an aromatic L-aminoacid decarboxylase [7,8]. There are two *Tph* genes: *Tph1* and *Tph2*. *Tph1* is expressed mostly in cells of the gut and is responsible for the production of peripheral serotonin [9]. *Tph2* is expressed exclusively in neurons of the brainstem and is responsible for the production of serotonin in the brain [8]. Moreover, serotonin does not cross the blood brain barrier; therefore it should be viewed from a functional point of view as two distinct molecules [7]. Brain-derived serotonin (BDS) acts as a neurotransmitter, while gut-derived serotonin (GDS) acts as a hormone and regulates a wide variety of processes [10]. The importance of serotonin in the regulation of bone mass is underscored by two clinical observations. First, depressed patients, that allegedly have low serotonergic tone, also have low bone mass [11]; and second, serotonin reuptake inhibitors (SSRI's) when taken chronically can either increase or more often decrease bone mass [12].

Our studies with loss and gain of function mutations of low-density lipoprotein receptor-related protein 5 demonstrated that GDS is a powerful inhibitor of osteoblast proliferation and bone formation that does not affect bone resorption [13]. Although correlative in nature, these studies showed that an increase in extracellular concentration of blood serotonin in patients on SSRI's may explain their often observed low bone mass phenotype [12]. However, the influence of this gut-bone axis on bone mass could not explain the increase in bone mass observed in another study with SSRI's [14]. In our quest to understand the serotonin regulation of bone mass in vertebrates we inactivated*Tph2,* the gene that catalyzes the rate-limiting step in the biosynthesis of BDS. The absence of serotonin in the brain resulted in a severe low bone mass phenotype affecting the axial (vertebrae) and appendicular (long bones) skeleton [15]. This phenotype was secondary to a decrease in bone formation parameters (osteoblast numbers and bone formation rate) and to an increase in bone resorption parameters (osteoclast surface and circulating Dpd levels) [15]. Hence, BDS is a positive and powerful regulator of bone mass accrual acting on both arms of bone remodeling despite accounting for >5% of total serotonin pool in the body it overrides the GDS regulation of bone mass [15].

While we were doing these studies we noticed, upon opening the abdominal cavities, that *Tph2*-deficient animals had a dramatic decrease in their adipose mass [15]. This prompted us to analyze in great detail their energy metabolism phenotype. The decrease in their fat mass was due, in part, to the fact that these mice ate less and spent much more energy compared to their wild type littermates [15]. This observation was not entirely surprising since serotonin is known to play important roles in many other physiological processes. However what caught our attention was the fact that the three most notable phenotypes of adult *Tph2*-deficient animals i.e., decrease in bone mass and appetite, and an increase in the energy expenditure are a mirror image of what is observed in mice that lack leptin [16,17].

**Figure 1.**
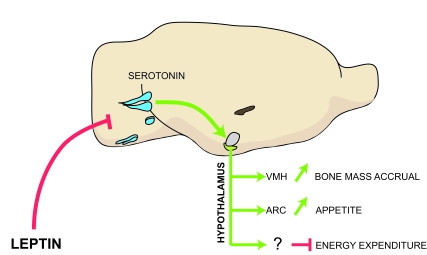
Model of the leptin-dependent central control of bone mass, appetite and energy expenditure. Leptin inhibits release of brainstem-derived serotonin, which favors bone mass accrual and appetite through its action on hypothalamic neurons. Serotonergic neurons are in blue; VMH, ventromedial hypothalamus; ARC, arcuate; VMH is in purple and arcuate is in green.

Three correlative experiments suggested that leptin might signal in the serotonin neurons to regulate some of its downstream functions. First, leptin receptor is expressed on serotonin neurons located in the raphe nuclei of brainstem and is functional [15]. Second, serotonin neurons project to the key hypothalamic nuclei responsible for the regulation of appetite, energy expenditure and bone mass [15]. Third, patients on SSRI's have been reported to have changes in their appetite and bone mass [12,18]. To explore that leptin may signal through the serotonin neurons to achieve these three functions, we inactivated leptin receptors in different nuclei of the hypothalamus or the serotonergic neurons of the brainstem [15]. Mice lacking *ObRb* either in *Sf1*-expressing neurons of the ventromedial hypothalamus (VMH) nuclei or in *Pomc*-expressing neurons of the arcuate (ARC) nuclei had normal sympathetic activity, bone remodeling parameters and bone mass; they also had normal appetite and energy expenditure, and when fed a normal diet, did not develop an obesity phenotype [19,20]. In contrast, mice that lack *ObRb* in serotonin neurons (*ObRb_SERT_*-/-) developed a high bone mass phenotype; they had a similar increase in appetite as *ob*/*ob* mice and had low energy expenditure. As a result, *ObRb_SERT_*-/- mice, when fed a normal diet, developed an obesity phenotype. These genetic studies demonstrated that leptin signals in the serotonin neurons of the brainstem to regulate, to the most part, bone mass, appetite and energy expenditure (Figure 1).

The demonstration that leptin-dependent central control of bone mass, appetite and energy expenditure occurs through its ability to inhibit serotonin production raised questions about the location and identity of serotonin receptors on hypothalamic neurons mediating these function. Double fluorescence in situ hybridization and nuclei-specific gene inactivation experiments revealed that serotonin promotes bone mass accrual through Htr2c receptors expressed on the VMH nuclei, while appetite through Htr2b and Htr1a receptors expressed on ARC nuclei of the hypothalamus. Further analysis revealed that Htr2c is upstream of the sympathetic center of the brain while Htr1a and Htr2b achieve their functions on appetite through modulation of melanocortin signaling (Figure 1).

In summary, these studies provide new insights into the central control of appetite and bone mass accrual and they identify serotonin as a focal point in the leptin-dependent common central control of bone and energy metabolisms.

## Acknowledgement

This work was supported by NIH grants (VKY, GK) and a Rodan fellowship from IBMS (VKY).
